# Her Heart's Mistake

**Published:** 1889-07-13

**Authors:** E. D. Cumming

**Affiliations:** Author of "An Ordeal by Silence"


### Her Heart's Mistake.

By E. D. Cumming, Author of "An Ordeal by Silence."

*(Continued from page 220.)*

### Chapter XIII.—Kate's Memory Fails Her.

Captain Phelps and Mr. Warren were lying in their long chairs in the verandah of the former's bungalow smoking earnestly and silently. A very close friendship had sprung up between these two, and their intimacy had reached that desirable stage when they could spend hours together, content with each other's society, without feeling it necessary to exchange a remark. Neither was in a communicative frame of mind this morning, and they lay absorbed in contemplation of the blue clouds which rose from their mouths, and swirled upwards to the bamboo rafters.

Mr. Warren was debating within himself whether he might not venture to make a call at the corner bungalow now that Dr. and Miss Harrison were re-established there. For they had spent a fortnight with Mrs. Macdonald whilst their home had been in the hands of a small army of men who had scoured and cleaned it after the visitation of cholera. He had not had an opportunity of speaking to either father or daughter since Mrs. Brent's death, and was not sure whether the Doctor would care to see visitors just yet. Though he lived at the club, and never lacked society, he did not find it so much to his taste as the companionship of Dr. Harrison. The ladies of the station made a practice of going regularly to see Kate, and pass the long, lonely days with her; but he felt that he ought to await an invitation before he intruded upon them. Captain Phelps dreamed sweet day-dreams of a nameless somebody.

The two men spent an hour in sociable muteness, and then Warren threw away the stump of his cheroot and stood up to go. It was past eight o'clock, and having sent his pony away, he wanted to walk down to the club before the sun grew hot.

"Off?" enquired Phelps.

"Yes; I've got a lot to do after breakfast; bye-bye."

As he passed out of the compound, Miss Harrison rode up on Baroness and drew rein as she overtook him.

"Good morning, Mr. Warren," she said. "I have been hoping to see you for the last three or four days."

Warren raised his hat, and expressed his regret at not having met her before.

"I was going to ask you to come in occasionally in the evening to talk to papa," she said, "he feels my aunt's loss very much, and would, I know, like to see you sometimes, if you could spare an hour or so."

"I had been wondering whether I might venture to come soon, Miss Harrison," he replied, "and am glad you have asked me."

"I don't believe in the system of shutting oneself up with sorrow," said Kate. "I think it's best to be guided entirely by one's own feeling in these matters, within certain limits, of course. I should be very wretched, if people did not come in often."

"I have no doubt you find it difficult to pass the time," said Warren.

"I can always occupy myself in one way or another; but somehow I never feel in the evening that I have done more than kill the day, and that I have no other object for the next and the next."

"Your feeling is that you have practically done nothing?"

"And make her livelihood by it?"

"Yes, a sense of general uselessness: that the only thing I can do with time is to kill it, when I might use it."

"You look after the house, do you not, and keep an eye on domestic affairs?"

"Yes, of course; but that doesn't take up one hour of the twenty-four."

"What else do you find to do?"

"Well, I practise for perhaps three or four hours, not because I like practising, but for the simple reason that I must do something. Then, when anyone comes in, I take up a piece of work of some kind. Unfortunately, my talents don't lie in a dressmaking direction, or I might be able to be useful at least to myself; so I keep my fingers out of mischief by working at something which at most will be pretty—and useless—when it's done. When that's finished I begin something else—often before it's finished if I get tired of it. One can't take much interest in work whose only *raison d'être* is to afford work. Do you think so?"

"No, I don't see how it's possible."

"When I am tired of working, if I am alone I take up a book. I do more reading than anything else; but so far I can't see that I am very much the better of it. I keep up my French and German, too, in hopes that *they* may be turned to account in killing time some day. I dare not hope they will ever be of any real practical use."

"You will discover their value if you happen to be travelling on the Continent."

"If ever George and I travel in Europe we shall only do so for pleasure. In other words, to kill time."

She answered with a little laugh, which had a ring of cynicism in it, and Warren felt that she had been speaking in earnest.

"Has your ambition to be useful ever taken any definite shape, Miss Harrison?" he asked.

"Not yet; I only began to think about these things lately, and I don't know which way to look to find what I want."

"Are the social boundaries beginning to look too narrow?" asked Warren, with a smile.

"I hardly know yet; I cannot help thinking they are wide enough for my powers. I am not one of the clever people," she added, with a laugh.

"Have you no ambition beyond your present sphere?" she asked, after a pause.

"I had once," replied Warren, "when I was two or three-and-twenty; they were so large that the world wasn't big enough for them. A fellow always begins like that; now they are only idle dreams."

"I don't know why they should be."

"I think when a man has passed thirty without discovering the road to their realisation they are apt to grow misty and dim. I have found it the case, at all events."

Nothing further was said on either side, and they remained silent until they came to the Harrison's bungalow, where Kate stopped.

"Good morning, Mr. Warren," she said, stooping from her saddle to shake hands with him. "Don't forget that we shall expect you any evening after half-past eight."

Warren bade her adieu and continued his walk to the club, thinking over the conversation they had just had. This was one of the women who could see beyond the prescribed circle, though she did not know it; he could not imagine Kate doing anything to gain a reputation of fastness, or anyone calling her unduly strong-minded. She was before all things a womanly girl; not brilliantly clever, perhaps, but with her full share of intelligence and common sense. Was she an exception to the rule he had so confidently laid down that night? Was she going to teach him that a clever girl could find ample scope for her abilities, and craving for useful work, within that confined circle? She was dissatisfied with the life she was leading, as his theory asserted all women above the average must be. He could not conceive her engaging in anything which would not become a woman; and he felt sure that she would find means to gratify her wish to be of use in the world. How would she do it? Warren felt that he had begun to take an interest in Kate Harrison, and determined that if he could help her he would, though how he was to do so he had no very clear idea. He did not fail to take advantage of her invitation to come and sit with her father in the evenings. He went to the corner bungalow that night, and continued to make a practice of doing so two or three times a week, to smoke and chat with the Doctor. Kate seldom joined in their conversations; she generally passed the time reading, or in writing to George Bairdsley. She had written to him more frequently of late. More than once she had dwelt at length upon her yearning to "use time instead of killing it"; but George did not seem able to enter into her feelings, and referred to them so lightly in his replies that she could not fail to see that he did not understand or sympathise with her aspirations. It was a disappointment to her, and that vague sense that they were drifting apart, that she was losing touch with him, was slowly and imperceptibly gaining ground. The only remedy she could suggest to herself was to write more often, and that she adopted, vanquishing the dawning doubts by nursing her wish to see him again. She made a point of writing for an hour or so every evening, so that her letter when despatched added, as it were, another page to the diary, through which George Bairdsley saw her life. He learned to know her more thoroughly through her letters, and their greater frequency only served to strengthen the belief he cherished that her whole soul was wrapped up in him.

One evening, the driving rain, which beat into the verandah, forced the two gentlemen to seek refuge in the room where Kate was reading, and she laid down her book to join in their conversation. It might have been her presence that brought up the topic, but whatever the cause, it was not long before Warren and the Doctor were engaged in discussion upon "women's work." She listened eagerly to the young barrister's forcible arguments in favour of the weaker sex being encouraged to take a greater share in the real business of life; and saw with a degree of pleasure that was unaccountable to her than her silent approval gratified and stimulated him. Dr. Harrison did not agree with the opinions Mr. Warren advanced; he had no doubt that women were mentally capable of higher things, but he was old-fashoned enough to prefer that they should centre their interests in the drawing-room and the nursery, where he thought their influence could be exercised with the greatest effect. Warren fully admitted that a wife and mother would best fulfil her duties by devoting herself to domestic concerns; that was a point no sane person would dispute. But did not the Doctor think that those women who had no such home ties might, with advantage to all, be permitted to seek independence? Dr. Harrison could not think it would be advisable; he looked upon a woman's mission as a purely domestic one; marriage was, to ninety-nine girls out of a hundred, much what his profession or business was to a man, and for his part he should be sorry to see them encouraged to seek independent paths. To allow them to compete with men, he thought, would unsex them and throw society off its balance. Matrimony should be their first object, and if they achieved it they would find the duties it imposed quite sufficient to keep them occupied. Then what would the Doctor suggest for the thousands of women who did not marry; it was on their behalf that social reform was required. The Doctor was inclined to think that very many of those women had simply missed what he considered their vocation in life through striving after the unattainable, and had themselves to blame for their position. Kate wanted to know what her father meant by the unattainable; did he mean that they allowed opportunities of marrying to pass in the hope of receiving better ones? Yes, to a small extent that was what he meant; but he had more in mind the restless disposition which led women to grow dissatisfied with their own sphere and encroach on that of men. He thought men fought shy of such women as a general rule, and he wasn't surprised at it. Warren did not press his views any further, and soon after the subject had been dropped, the Doctor disappeared into his own room, leaving him alone with Kate. Neither of them spoke for a few minutes, but at length the young lady touched upon a remark her father had made.

"I don't like the idea of marriage being considered a woman's business or profession, Mr. Warren. Do you?"

"I think there's much truth in it, in the present order of things; but it sounds a hard, worldly way of speaking. Your father doesn't mean it quite literally, Miss Harrison."

"I know that," laughed Kate, "but I prefer to think that hearts have more to do with matrimony than heads."

"Heads ought to have a good deal of say in the matter."

"I suppose they ought, but I can't accept the suggestion that marriage should be regarded as papa put it."

"If that vocation—to use the Doctor's expression—is denied a woman, it appears to me a hardship that she should not be allowed to seek an alternative one."

"Yes; in certain paths of life."

They had wandered out on to the verandah as they talked; the rain had ceased, and the clouds had vanished, leaving the sky to the rule of the brilliant moon, which bathed the bungalow and its surroundings in the softest shadowy light. Kate stood, with her hands clasped behind her, looking out, and Warren leaned against the railing, glancing in admiration from time to time at the face whose beauty was enhanced by its grave intelligence. He was beginning to wonder what manner of man Captain Bairdsley was to have captivated such a woman as this. He had heard a good deal about him from his brother officers, but nothing which proved his peculiar fitness, above all other men, to be Miss Harrison's husband.

He remained talking with her for nearly an hour longer, growing more and more interested in her as he did so. His syce had brought his pony round to the door, and after waiting below the porch unnoticed for a while, had taken up his position under a tree, where he was now squatting with the bridle in his hand, emitting vigorous coughs once a minute to remind his master that he was waiting. He succeeded in forcing himself upon his attention at last, and Warren looked at his watch in surprise. It was after eleven. He stopped in the midst of the remark he was making to his companion, and bade her good night. Kate watched him mount his pony and ride off, her mind full of the subjects they had been discussing that evening. She waited until the sound of hoofs died away before she turned towards her room. Suddenly she uttered an exclamation and stood still. She had, until this moment, completely forgotten her usual letter to George Bairdsley.

*(To be continued.)*

